# Nonadiabatic dynamics in multidimensional complex potential energy surfaces†

**DOI:** 10.1039/d0sc04197a

**Published:** 2020-09-07

**Authors:** Fábris Kossoski, Mario Barbatti

**Affiliations:** Aix-Marseille Univ, CNRS Marseille France fabris.kossoski@univ-amu.fr mario.barbatti@univ-amu.fr

## Abstract

Despite the continuous development of theoretical methodologies for describing nonadiabatic dynamics of molecular systems, there is a lack of approaches for processes where the norm of the wave function is not conserved, *i.e.*, when an imaginary potential accounts for some irreversible decaying mechanism. Current approaches rely on building potential energy surfaces of reduced dimensionality, which is not optimal for more involving and realistic multidimensional problems. Here, we present a novel methodology for describing the dynamics of complex-valued molecular Hamiltonians, which is a generalisation of the trajectory surface hopping method. As a first application, the complex surface fewest switches surface hopping (CS-FSSH) method was employed to survey the relaxation mechanisms of the shape resonant anions of iodoethene. We have provided the first detailed and dynamical picture of the π*/σ* mechanism of dissociative electron attachment in halogenated unsaturated compounds, which is believed to underlie electron-induced reactions of several molecules of interest. Electron capture into the π* orbital promotes C=C stretching and out-of-plane vibrations, followed by charge transfer from the double bond into the σ* orbital at the C–I bond, and, finally, release of the iodine ion, all within only 15 fs. On-the-fly dynamics simulations of a vast class of processes can be envisioned with the CS-FSSH methodology, including autoionisation from transient anions, core-ionised and superexcited states, Auger and interatomic coulombic decay, and time-dependent luminescence.

## Abstract 2

Despite the continuous development of methods for describing nonadiabatic dynamics, there is a lack of multidimensional approaches for processes where the wave function norm is not conserved. A new surface hopping variant closes this knowledge gap.

## Graphical abstract

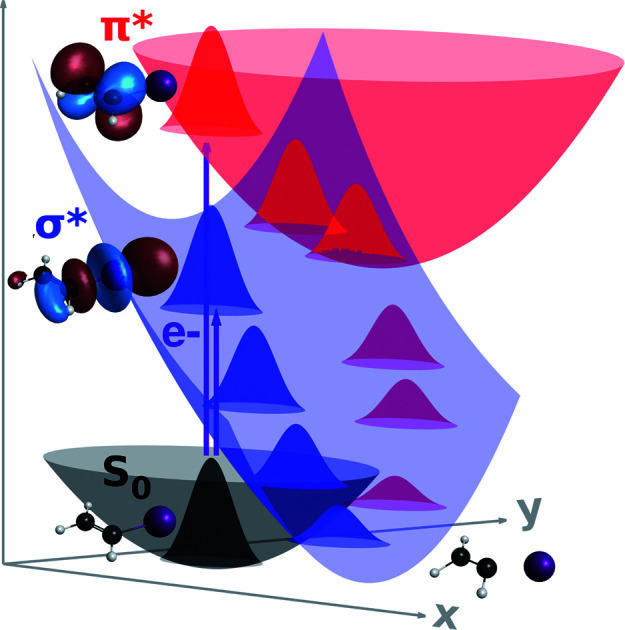

## 1 Introduction

Computational methods for nonadiabatic dynamics simulations comprise a fundamental theoretical tool for surveying the relaxation of electronically excited molecules. Basically, an approximate solution for the electronic problem provides the potential energy surfaces (PESs) that guide the nuclear dynamics. At the same time, the coupling between the motion of light and heavy particles is achieved in one of several ways, depending on the particular mixed quantum-classical approach. Applications of these methodologies are countless, as can be attested by three recent reviews of the topic.^1–3^

Despite their success, the current formulations cannot be applied to open quantum systems, where the set of discrete states of the system interacts with a continuum of scattering states.^4,5^ This class of problems requires the use of non-Hermitian quantum mechanics, which is a vast ever-growing topic on itself,^5,6^ going beyond the molecular dynamics subject that concerns us here. But in short, the effect of the continuum of states can be modelled with imaginary potential terms, which mimic the decay from the discrete states and account for the decrease of the system wave function. Within the non-Hermitian formalism, the effective Hamiltonian eigenstates become resonances, with complex-valued energies *E*_r_ − i*Γ*/2, where *E*_r_ is the resonance energy and *Γ* the resonance width, whose inverse gives the lifetime of the metastable state.^5^ For example, in the context of molecular systems, this usually means localized discrete states, which are embedded into a continuum of autoionizing scattering states. Then, the PESs that drive the dynamics also become complex-valued, and *Γ* represents the rate of electron autoionization (or autodetachment).

Even though there are implemented methodologies based on quantum wave packet propagation on coupled and complex PESs,^7–10^ in practical terms, they are restricted to problems with few degrees of freedom, hence limiting their potential applicability. In another direction, we have presented a computationally feasible formulation that accounts for the multidimensional and complex character of the PESs,^11^ yet it missed the inclusion of nonadiabatic effects. It is also worth mentioning recent theoretical efforts in search of critical points in complex PESs.^12,13^ Counting with quantum chemistry methods adapted with complex absorbing potentials,^14^ it is now possible to locate minimum energy crossings between anion and neutral states,^12^ as well as exceptional points in complex PESs^13^ (which are the complex analogous of conical intersections of real PESs^15,16^).

Despite these relevant developments, there is still not a computationally feasible method for full multidimensional molecular dynamics simulations that account for both non-Hermitian and nonadiabatic effects. Here, we propose a novel theoretical methodology that fills this important gap. It comprises a generalization of the trajectory surface hopping (TSH) method^1^ to the case of complex-valued PESs, while employing the popular fewest switches surface hopping (FSSH) flavor^17^ for the hopping probabilities. As such, we name it complex surface fewest switches surface hopping, or CS-FSSH for short. Our main goal is to present the method and establish its potential for performing dynamics simulations of electronically metastable molecular states. We have first validated it by comparing to numerically exact quantum dynamics simulations on model analytical PESs. Then, we demonstrate its potential by surveying the dynamics of a resonant state in an actual molecular system. We mention an independent development^18^ where the FSSH approach was applied to complex-valued PESs. There, the authors have employed a one-dimensional model to describe cavity losses in the polaritonic dynamics of azobenzene.

Various kinds of molecular states can undergo irreversible decay to the continuum, and thus can be modelled within a non-Hermitian formalism. Transient anions (shape resonance) are formed by direct attachment of free electrons to a neutral species, which then decay either by electron autodetachment (such that the inverse of *Γ* provides the detachment lifetime) or by molecular dissociation. The latter relaxation channel, dissociative electron attachment (DEA), is ubiquitous in molecules of various types, even though its underlying working mechanisms are far from understood.^19^ Transient anions can also be populated by photoexcitation of an electronically bound anion,^20,21^ possibly accessing different regions of the PESs and leading to distinct decaying channels. Related processes include dissociative recombination^22^ and associative detachment, the latter being the inverse process of DEA. Metastable electronic states that lie above other ionization thresholds can also decay by electron detachment, of which we cite multiply charged anions,^23^ superexcited,^24,25^ core-excited and core-ionized states.^26^ The latter kind of state can also decay by two-particle processes such as Auger^27^ and interatomic coulombic decay.^28,29^ Furthermore, metastable states can be formed in bimolecular collisions, which include charge transfer in atom-molecule,^30^ ion-molecule collisions,^31^ and collisional detachment.

From all the above-mentioned processes that can be formulated in terms of a complex-valued Hamiltonian, we have chosen to survey a DEA reaction as the first application of our proposed methodology. DEA is known to play important roles, to a greater or lesser degree, in DNA damage induced by ionizing radiation,^32,33^ in the action of radiosensitizing drugs,^33,34^ in the chemical evolution of interstellar media^35^ and planetary atmospheres,^36,37^ in plasma technologies,^38^ among many other examples.^19,39^ Of particular relevance herein, low energy electrons can very efficiently promote DEA reactions in halogen-containing unsaturated compounds. This process is believed to account for the radiosensitization properties of modified nucleobases,^34,40,41^ and also for the degradation of persistent halogenated compounds.^42^ The general picture is that electron attachment forms a π* shape resonance (associated with the unsaturated region), which undergoes efficient vibronic coupling to σ* resonances (dissociative along the carbon–halogen bond).^43–46^

While this π*/σ* mechanism is widely accepted to take place in halogenated species, being often evoked to interpret experimental data,^40,41,47^ it has never been theoretically demonstrated, to the best of our knowledge. More importantly, though, the overall understanding about it is rather qualitative, as the underlying mechanisms that drive the reaction are not yet figured out. In particular, there is no information about the sequence of steps of the DEA pathway, its timescales, and the couplings among electronic states. On top of that, it is still not clear how DEA triggered by π* resonances can present cross sections extending over several orders of magnitude.

As a first application of the CS-FSSH methodology, we have investigated the low energy electron induced dynamics of iodoethene. The molecule is expected to display a prototypical π*/σ* indirect DEA mechanism,^46^ which should also be encountered in biologically relevant halogen-containing molecules.^40,41^ Our goals with this particular application are twofold. First, to provide theoretical evidence for the existence of the π*/σ* mechanism, and second, to unveil the underlying pathway that culminates in molecular dissociation. Some of our findings might start to shed some light on how DEA reactions take place in larger halogenated molecules.

## 2 Methodology

The construction of the CS-FSSH methodology follows the same steps of the usual FSSH approach.^17^ In short, nuclei are propagated classically on the real-valued PESs computed on-the-fly with electronic structure theory, while the electronic wave function is propagated with the time-dependent Schrödinger equation (TDSE) on the complex PESs. The FSSH prescription mediates the coupling between electronic and nuclear degrees of freedom.

Let us consider a complex-valued electronic Hamiltonian *H* = *H*^R^ + i*H*^I^, with a real component *H*^R^ and an imaginary component *H*^I^. The electronic wave function *Φ*(**r**,**R**,*t*) is governed by the TDSE1
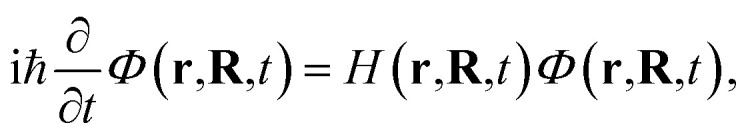
where **r** denotes the collective electron variables, and **R** those of the nuclei, with corresponding classical trajectory **R**(*t*) assumed to be known. Then, the time-dependent electronic wave function *Φ*(**r**,**R**,*t*) can be expanded in a set of orthonormal basis functions {*ψ*_*j*_}, which depend explicitly on **r** and implicitly on **R**(*t*),2
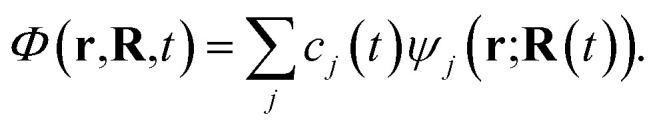


Introducing the above expansion into the TDSE, multiplying by 
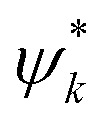
 and integrating in **r** leads to a set of coupled equations for the expansion coefficients *c*_*j*_,3
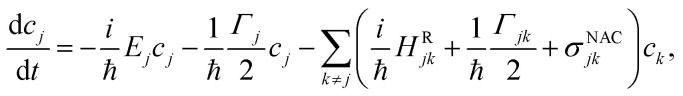
where the off-diagonal terms of the Hamiltonian are the diabatic couplings, 〈*ψ*_*j*_|*H*|*ψ*_*k*_〉 = *H*_*jk*_ = *H*^R^_*jk*_ − i*Γ*_*jk*_/2, while the diagonal terms, *H*_*jj*_ = *E*_*j*_ − i*Γ*_*j*_/2, provide the energies *E*_*j*_ and widths *Γ*_*j*_. We have introduced *σ*^NAC^_*jk*_ ≡ 〈*ψ*_*j*_|∂*ψ*_*k*_/∂*t*〉 = **F**_*jk*_·**v**, where **F**_*jk*_ = 〈*ψ*_*j*_|∇_**R**_|*ψ*_*k*_〉 are the nonadiabatic coupling vectors and **v** = d**R**/d*t* is the classical nuclei velocity. The deduction of eqn (3) is exactly the same as in usual TSH,^17^ with the key difference that here we acknowledge that energies and couplings might be complex-valued. Decoherence effects should still be included, and we do it with an adapted version of the simplified decay of mixing^48^ (equations are shown in the ESI, Sec. S1†).

The first term in the right-hand side of eqn (3) controls the phase of the amplitude *c*_*j*_, according to the energy *E*_*j*_. The second term accounts for the decrease of the complex amplitudes, whose rate is given by *Γ*_*j*_/ℏ. The populations *ρ*_*jj*_ are defined as the diagonal elements of the density matrix 
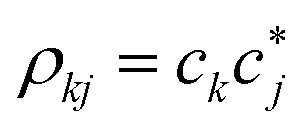
, and the total population *p* as the sum over the contribution of each PES, 
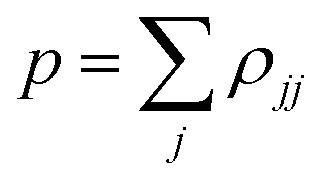
. In this way, the total population of each classical trajectory will not be conserved, but will lie between 0 and 1 instead. Finally, the third term in eqn (3) is responsible for the population transfer between states and has three contributions. Diabatic *H*^R^_*jk*_ and nonadiabatic *σ*^NAC^_*jk*_ coupling terms promote the direct interaction between states *j* and *k*. Additionally, the states may interact indirectly, *via Γ*_*jk*_, which represents a coupling through the continuum.

While the electronic wave function is propagated quantum mechanically, nuclei are assumed to be classical particles, which thus follow Newton's equations,4
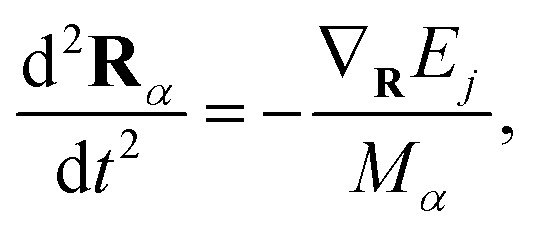
where a single PES *j* provides the forces, and *M*_*α*_ is the mass of nucleus *α*.

The decision of which is the current PES *j* that guides the nuclei dynamics is based on Tully's FSSH scheme.^17^ Within the adiabatic representation for the set {*ψ*_*j*_}, the formula for the hopping probabilities becomes5
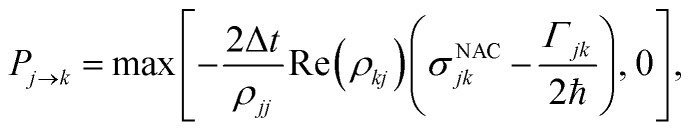


The above hopping formula differs from the usual FSSH one^17^ by the indirect coupling term *Γ*_*jk*_/2ℏ.

Whether a hopping event takes place or not is decided stochastically, by comparing the hopping probabilities to a random number between 0 and 1, at each time step of the TDSE. When the hopping algorithm indicates a hop to an upper PES, we must first ensure that total energy is conserved after the velocity vector is rescaled along the nonadiabatic coupling vector.^49^ If that is not possible, the hopping is said to be frustrated, and the dynamics resumes on the same PES.

Once the dynamics simulation is finished, any time-dependent observable of interest *ŝ*(*t*) can be computed by performing a weighted average over the contribution from each trajectory, as6
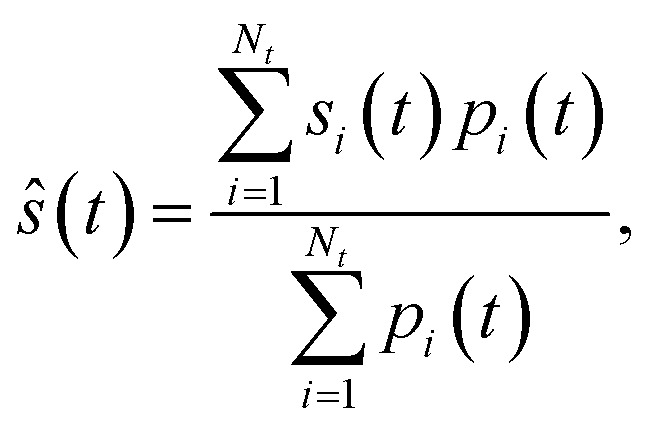
where the sum runs over a total of *N*_*t*_ trajectories, *s*_*i*_(*t*) represents the observable and *p*_*i*_(*t*) the total population, for trajectory *i* and at time *t*. While in usual FSSH, statistical averages are performed with the same expression, there is a key conceptual difference to the CS-FSSH methodology. In the latter, the total populations are different from 1, such that the contribution from each trajectory is weighted by the factor 
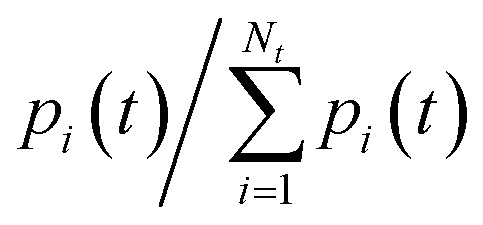
.

A limitation of the methodology is that the coupling between discrete and continuum states is unidirectional, *i.e.*, only hops from the former to the latter can take place, which is a reasonable approximation for most situations of interest. In the particular case of transient anions, it means that the molecule cannot recapture the detached electron. These so-called nonlocal effects should only be relevant at the lowest collision energies and for very broad resonances,^50^ which do not apply to our present case. The CS-FSSH methodology should also present the same limitations as in usual FSSH, concerning the lack of zero-point energy, interference effects, and quantum tunnelling. At the same time, the quality of the results is intimately related to the employed level of theory for describing the real and imaginary components of the PESs.

## 3 Validation against quantum dynamics

**Fig. 1:**
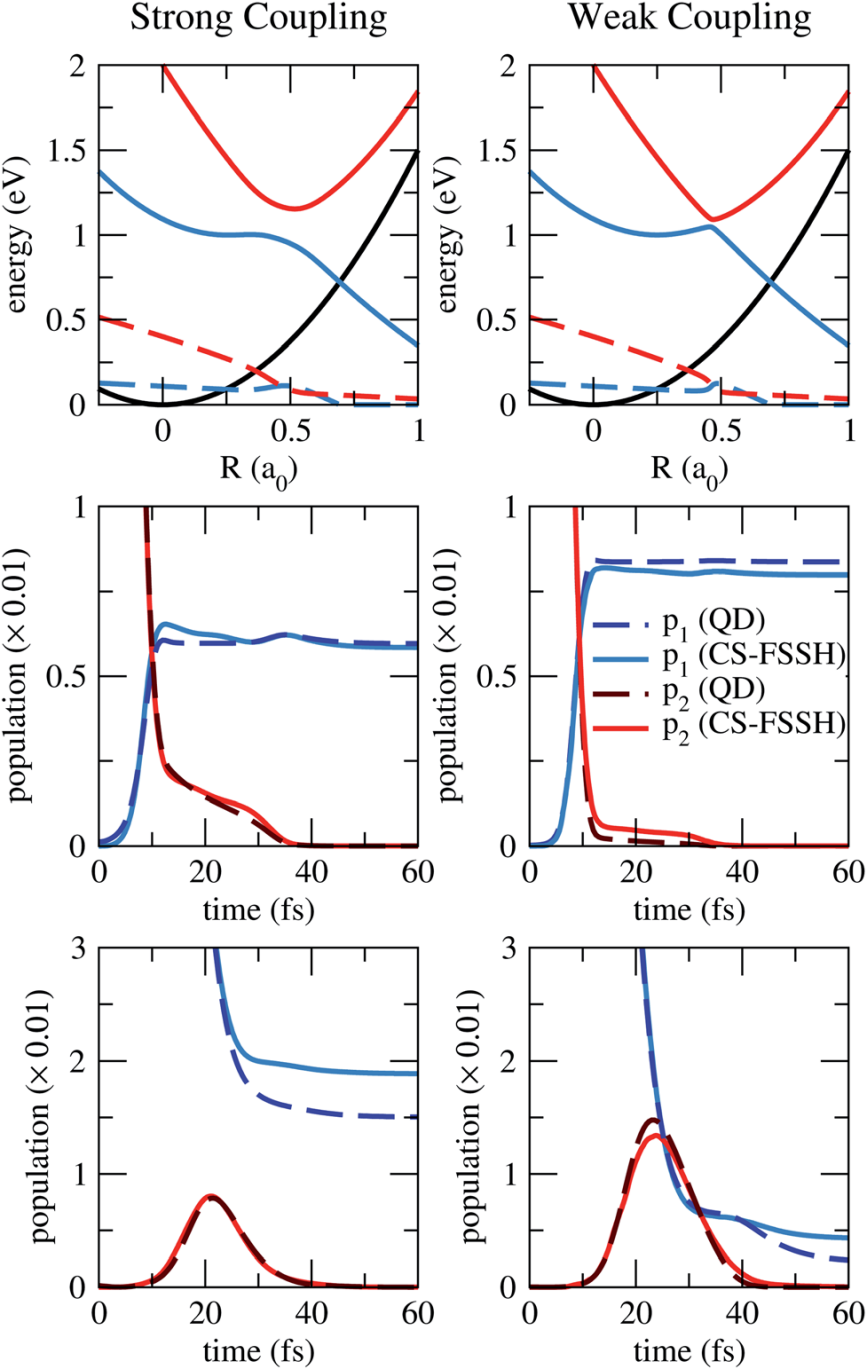
Results for the 2RHE PESs, for stronger (left) and weaker (right) diabatic couplings. On the top panels, energies (full lines) and widths (dashed lines) for the lower (blue) and upper (red) adiabatic resonant states, and for the state that defines the decaying threshold (black). Adiabatic populations are shown on the other panels, when the dynamics starts from the upper state (middle panels) or lower state (bottom panels). Populations for the lower (p_1_, blue) and upper (p_2_, red) states were obtained with the quantum dynamics (QD, darker dashed lines) and with the CS-FSSH methodology (lighter full lines).

Before employing the CS-FSSH methodology to a real molecular system, we have first benchmarked it against numerically exact quantum propagation results on model PESs, shown in Fig. 1.

Our diabatic potentials comprise two coupled resonances, one exponential and one harmonic (2REH model). An additional state is uncoupled and defines the decaying threshold where the resonance becomes a bound state, and was modelled as7
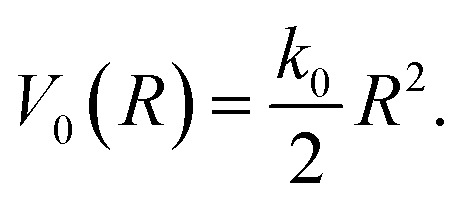


The exponential (dissociative) and the harmonic (non-dissociative) states are coupled, allowing nonadiabatic transitions between them in the adiabatic representation. Their real component diabatic potentials are given as8
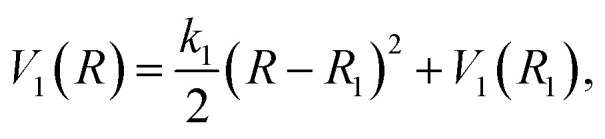

9 *V*_2_(*R*) = [*V*_2_(0) − *D*]e^−*αR*^ + *D*,

and they interact through the diabatic coupling

10 *V*_12_(*R*) = *V*_12_(*R*_c_)e^−*β*(*R* − *R*_c_)^2^^,

where *R*_c_ is the crossing point between *V*_1_ and *V*_2_. The coupling *via* the continuum was neglected, *Γ*_12_ = 0. Resonance widths were modelled as being proportional to the resonance energy,11
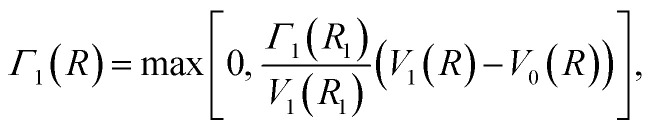
12
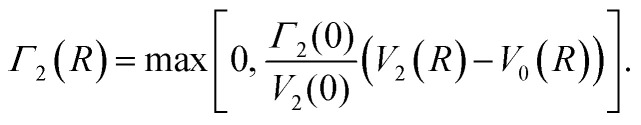


The vibrational ground state of *V*_0_ was employed as the initial wavepacket and as the sampling distribution for the ensemble of initial conditions.

The parameters of the 2RHE model, as well as the computational details concerning the CS-FSSH and quantum propagation, are presented in Sec. S2 of the ESI.† While different profiles for the PESs have been considered, the most important aspect to be assessed in our validation would be the strength of the coupling. Thus, we decided to compare results for two diabatic coupling strengths for the same set of diabatic PESs.

The adiabatic PESs and the evolution of the populations are shown in Fig. 1, as computed with both theoretical approaches. Along with the conventional population transfer between states, here the total population (sum of lower and upper populations, not shown) is reduced because of the coupling to the continuum. The main finding is that both the CS-FSSH methodology and the reference quantum propagation deliver quite consistent results for both weaker and stronger coupling strengths. Our methodology correctly reproduces the decay of the population and its transfer among the two resonant states.

We have surveyed four cases, for dynamics starting at the lower and upper states, and for weaker and stronger couplings. In all of them, the initial population drops by around two orders of magnitude, due to the positive resonance widths (notice the scale of the *y*-axis in Fig. 1). Then, population transfer takes place, which is more pronounced in the case of a weaker diabatic coupling, as would be expected. In the meantime, the population keeps decaying before the resonance becomes a bound state (blue-black crossing). Therefore, when dynamics starts in the upper state, the final total population is larger for weaker diabatic couplings. In contrast, a stronger coupling minimises the decay of population when the lower state is initially populated. Finally, the trajectories (or the wavepacket, for the quantum propagation) eventually dissociate along the lower PES, and the total population stabilises.

When starting at the lower state (bottom panels of Fig. 1), the final total population is somewhat superestimated in the CS-FSSH dynamics (with respect to the quantum results) for both coupling strengths. This effect is probably related to the presence of frustrated hoppings: the trajectory should hop to the upper state and lose an extra population, but instead, it remains on the lower one, where population decay stops shortly after. This issue could be minimised with alternative prescriptions when a frustrated hopping is encountered^51^ or with methods that impose energy conservation only at the ensemble level.^52,53^

## 4 Application to a realistic case

### 4.1 Computational details

For the dynamics propagation, both anion and neutral electronic states of iodoethene were described at the multireference configuration interaction (MRCI) level of theory. Two roots have been computed for the anion, which in the neutral equilibrium geometry corresponded to a lower-lying 
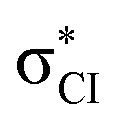
 state (σ* for short) and a higher-lying π* state. At other geometries, they mix their diabatic characters, and we refer to the lower and upper states of the anion. Inspection of the orbitals and energies along the trajectories confirmed that our computational protocol for the electronic problem avoided undesired pseudo-continuum states.

The resonance widths were modelled based on the *ab initio* electron scattering calculations, performed with the Schwinger multichannel method.^54^ Additionally, these calculations have corroborated the MRCI results, as the same valence anion states were obtained in both types of calculations.

The dynamics simulations were performed with a development version of the Newton-X package,^55,56^ interfaced with the Columbus package.^57^ Further details about the bound state, scattering, and dynamics calculations can be found in Secs. S3.1–S3.3 of the ESI.†

It is worth emphasizing that the CS-FSSH methodology is not restricted to this particular choice of methods for solving the electronic problem. It could be combined with any methodology that provides the required ingredients for the dynamics (energies, nuclear gradients, nonadiabatic couplings and resonance widths).

### 4.2 Modeling the complex PES

While the real-valued PESs were computed on-the-fly with the MRCI method, for the imaginary component, we have employed a model that combined scattering and bound state results.

First, some test dynamics simulations on real-valued-surfaces revealed which were the most active coordinates upon electron attachment. Based on that information, *ab initio* electron scattering calculations were performed for a couple of selected geometries, which provided resonance energies and widths. A cubic interpolation for this set of results provided our model for *Γ*_*j*_ as a function of the corresponding energy *E*_*j*_. Their profiles and the fitting parameters are shown in Fig. S1 and Table S2 of the ESI.† At each time step of the simulation, resonance energies were obtained from the bound state calculations (MRCI), which fed the model that provided the instantaneous widths.

In this first application, we neglected the nondiagonal terms *Γ*_*kj*_, which couple the two anionic states through the continuum. This contribution should be much smaller than the direct coupling *via***F**_*kj*_. Qualitatively, the former kind of coupling means the extra electron undergoes two hops (discrete to continuum, and then to discrete). This should be much less likely than the latter kind, which involves a single hop (discrete to discrete).

Furthermore, we have introduced a systematic correction to the real-valued PESs, based on the energies obtained with the scattering calculations. Additional details about this correction and the construction of the width model are given in Sec. S3.5 of the ESI.†

### 4.3 Electron-induced dynamics

**Fig. 2:**
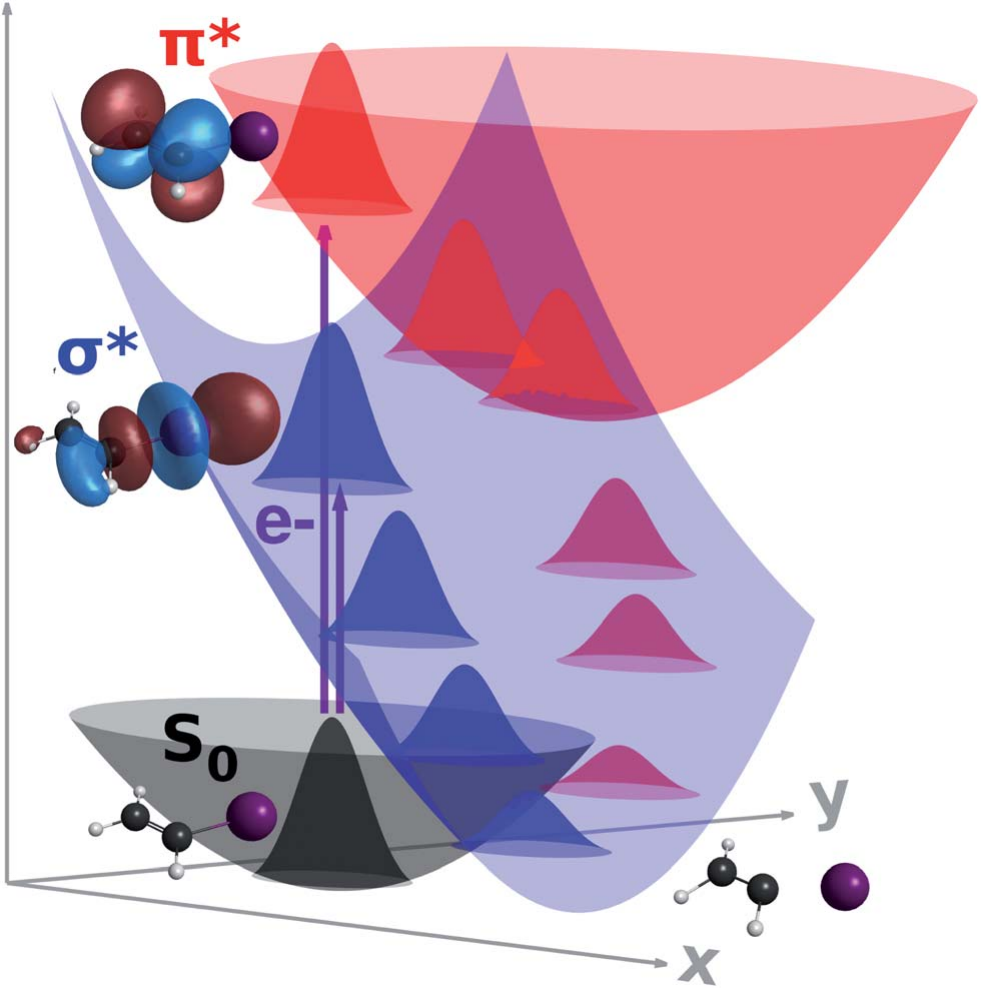
Schematic PESs for the neutral ground state (grey), and diabatic σ* (blue) and π* (red) anion states of iodoethene, along dissociating (*x*) and coupling (*y*) coordinates. Electron attachment into the σ* state promptly induces cleavage into the iodine ion, whereas a π*/σ* coupling offers a route for dissociation when the π* state is initially formed.

In the previous subsections, we presented the crucial computational aspects of our calculations and a practical way of computing *Γ*_*k*_ on-the-fly, which is used with the usual real quantities to integrate eqn (3). In the present subsection, we discuss our main findings for the electron-induced dynamics of iodoethene, whose dissociation mechanisms are schematically depicted in Fig. 2.

**Fig. 3:**
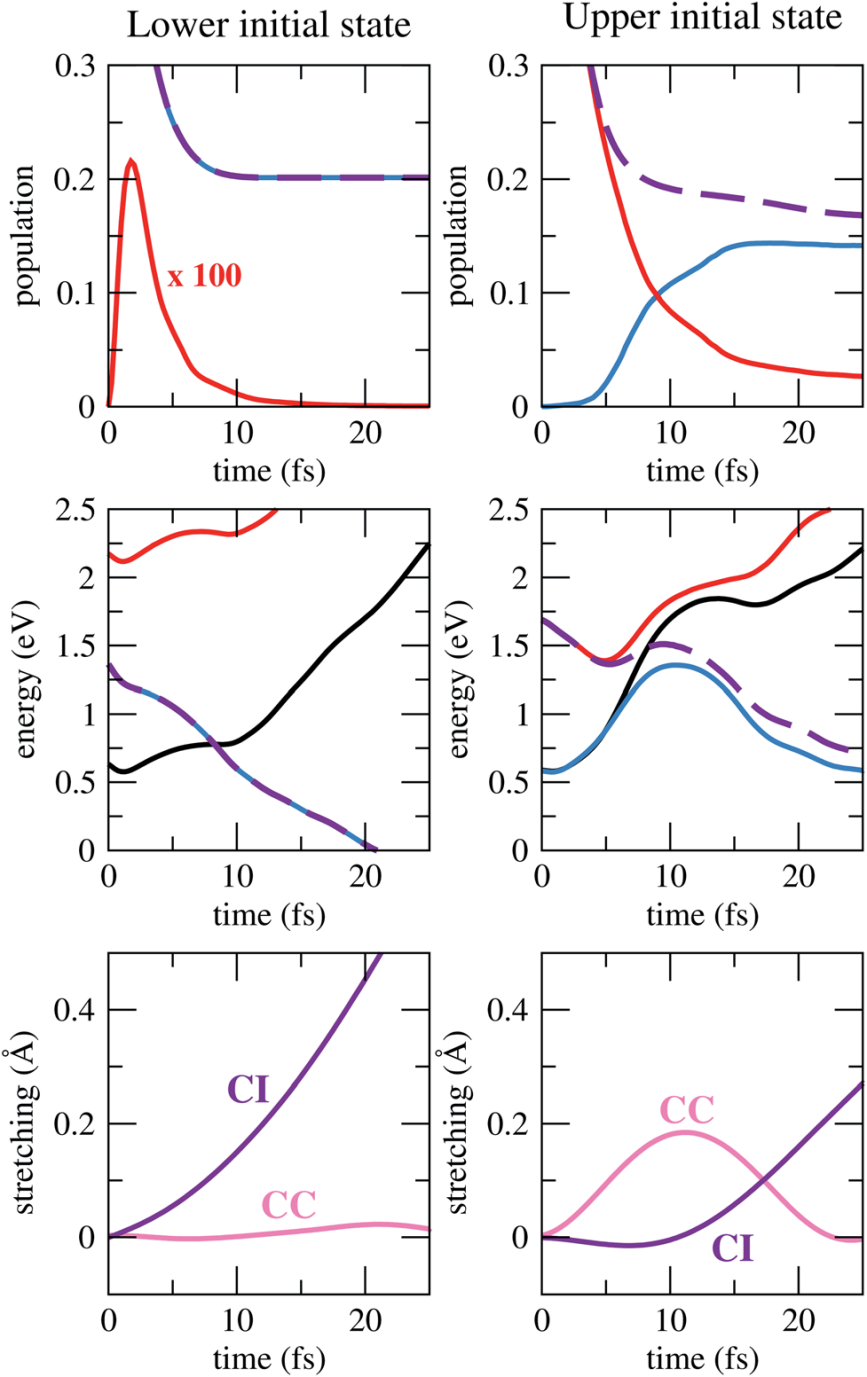
Results for dynamics starting at the lower (left) and upper (right) anion states. On the top panel, lower (blue), upper (red) and total (purple) adiabatic populations. On the middle, lower (blue), upper (red) and average (purple) anion energies, as well as average neutral energies (black), where 0 eV corresponds to the neutral at its equilibrium geometry. And on the bottom, ensemble average of the C–I (purple) and C=C (pink) stretching from the neutral equilibrium geometry.

Key observables from the simulations are presented in Fig. 3. Formation of the σ* anion state (left panels) is followed by a significant loss of population due to electron autodetachment, but also by rapid stretching of the C–I bond. This stretching, in turn, provides stability against detachment, until the resonance becomes an electronically bound state (at 8.3 fs in the ensemble average, see purple-black curve crossing). From there, cleavage of the bond resumes and the iodine ion is formed. The process is barrierless, as the ensemble average anion energy (which virtually always coincides with the lower state energy), decreases monotonically. At longer times, the total population stabilises at 0.20.

At electron impact energies around 1 eV, the π* resonance is formed (Fig. 3, right panels), and its relaxation is considerably more involved, yet extremely fast. Within 5 fs, the C=C bond is stretched (which works as the tuning coordinate), and the hydrogens move out of the molecular plane (coupling coordinate), while electron autodetachment reduces the total population to around 0.25. These geometrical changes bring the system close to a conical intersection, where several trajectories hop to the lower PES, mostly between 5 and 15 fs. This feature can be seen in the profile of the average anion energy, which initially matches with the upper state energy, and later approaches the lower one. Meanwhile, the anion changes its character from π*, to a mixed π*/σ* and finally to σ*. Once the latter dominates, the dynamics unfolds as described before, culminating into dissociation of the iodine ion.

In addition, strong vibrational excitation of the C=C mode takes place. Between 5 fs and 15 fs, the anion becomes electronically stable for most of the trajectories, such that the average ensemble population decreases much more slowly than in the beginning. The anion takes an average of 8.3 fs to become more stable than the neutral, which coincidentally is the same as when the σ* initiates the dynamics. Trajectories that missed the first opportunity to hop to the lower PES eventually do so, but with increasingly smaller populations as time passes by, since they still remain on regions of the PES with positive resonance widths. The total population finally converges to around 0.17.

An important finding concerns the observation of an energy barrier when the π* resonance triggers the dynamics (see middle right panel of Fig. 3). Since it represents an ensemble quantity, it does not mean that every trajectory encounters a barrier, but rather that it manifests itself in an average way. In the present case, the top of the barrier lies below the initial energy, and therefore it does not hinder the dissociation pathway. This result is consistent with what has been discussed for the similar chloroethene molecule,^13^ which is believed to display an analogous π*/σ* DEA pathway.^46^ In this previous study, however, the discussion was supported by one-dimensional PESs interpolated between critical points, including the minimum energy crossing for the relevant anion states and between anion and neutral. In contrast, our conclusion about the energy profile has been obtained dynamically. Importantly, we have also found that between 5 fs and 10 fs, the ensemble neutral energy rises even faster than the anion energy, such that the resonance energy actually decreases and drops to zero before the top of the barrier is reached. If that were not the case, the presence of the barrier would leave extra time for electron autodetachment, thereby disfavouring decay by DEA.

**Fig. 4:**
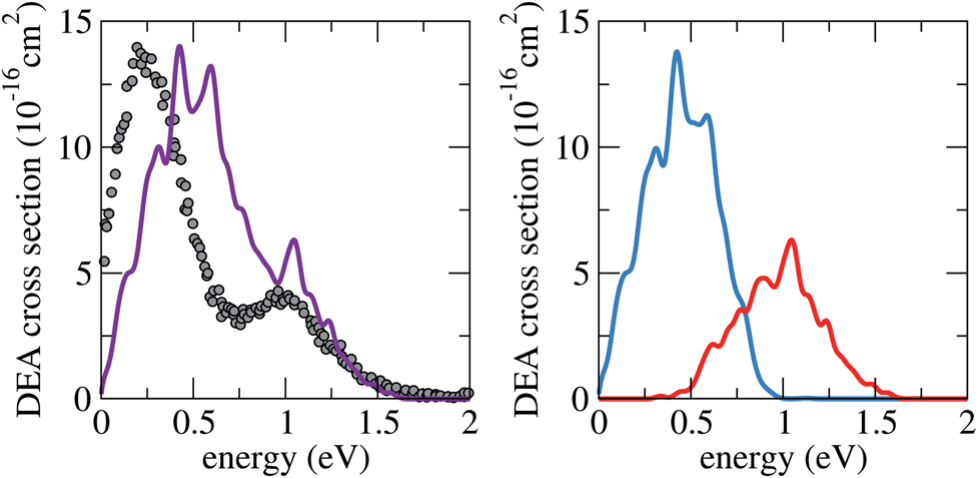
Calculated DEA cross section for iodoethene (purple), when starting from the lower (blue) or the upper (red) anion states, compared to available experimental data (grey dots).^46^

In Fig. 4, the computed DEA cross sections (see Sec. S3.4 in the ESI†) are compared to the single available set of experimental data.^46^ The latter was not presented in absolute units, and as such the data has been normalised to our most intense peak. The main finding from this comparison is that our calculations are able to reproduce the relative contribution of each state to the DEA cross section. The difference in magnitude is not related to the final populations, which are quite similar (0.20 and 0.17). Instead, it is simply due to the 1/*E* kinetic factor that appears in the DEA cross sections expression (eqn (S6) in the ESI†).

Part of the observed discrepancy of around 0.25 eV for the position of the first peak can be traced back to inaccuracies in the scattering calculations (details in the ESI, Sec. S3.5†). Additionally, nonlocal and zero-point energy effects (not encompassed in the methodology) could play a role, given the proximity of this resonance to the detachment threshold.

## 5 Conclusions and outlook

This contribution provides a novel and general theoretical framework for describing multidimensional nonadiabatic dynamics of molecular systems where irreversible decaying mechanisms take place. The CS-FSSH methodology is a natural generalisation of the FSSH method for complex-valued potential energy surfaces. As such, it assimilates the underlying concepts of an ensemble of classical trajectories and hopping between PESs, but here the total population may decay with time, and the hopping probability is modified by imaginary couplings terms. The comparison with respect to quantum dynamics results for our one-dimensional 2RHE model is outstanding. Both theoretical approaches provided quite similar behaviours for the populations, in both strong and weak couplings regimes. The method has been implemented into a developing version of the Newton-X package, which is going to be freely available to the community in a future release of the code.

As a first realistic application of the CS-FSSH methodology, we have surveyed the dissociation of iodoethene induced by low-energy electron attachment. Our simulations provided a detailed picture of the underlying relaxation mechanisms of the transient anion states. Formation of the σ* resonance promptly induces cleavage into the iodine ion. In contrast, DEA from the π* resonance was found to be intrinsically multidimensional, as it first relaxes by elongation of the C=C bond and out-of-plane vibrations, which promote the coupling to the σ* state and C–I bond breaking. This result is the first theoretical confirmation of the long proposed π*/σ* coupling mechanism in DEA reactions of halogenated molecules. The change of character between anion states takes place mostly between 5 fs and 15 fs, while electronic stability is reached within 10 fs on average. Furthermore, the dissociation pathway encountered a low energy barrier, already past the autodetachment region, which therefore did not quench the DEA channel. However, the presence of such barriers might play a role in explaining the large variations in the efficiency of electron-induced reactions. As a final validation of the methodology and calculations, the shape of the computed DEA cross sections agreed quite well with the available measured data.

The exploitation of the methodology in the context of transient anions is expected to push forward our understanding of the very rich processes induced by low-energy electrons. This, in turn, would provide important insights to various fields, ranging from astrochemistry, to radiobiology and material sciences.

In addition, the methodology could also be employed to simulate the dynamics of molecular systems that present arbitrary decaying mechanisms to a continuum, which would be modelled by imaginary potentials. As stated in the Introduction, this includes Auger and interatomic coulombic decay, and electron detachment from neutral super-excited and core-excited states and transient anion states, the latter being formed by electron impact, photoexcitation or bimolecular collisions. Dynamics simulations of these processes might become feasible by employing the CS-FSSH methodology in conjunction with proper modelling of the decaying widths. This would be accomplished with approaches that combine different types of calculations, as we have done here, with scattering and bound state methods. Alternatively, one could use adapted quantum chemistry methods that directly provide information about the widths.^14,58,59^

As one final potential application of the methodology, we put forward the idea that it could be employed to incorporate the possibility of radiative decay into nonadiabatic dynamics of excited states. Although there are available computational codes for mixed quantum-classical dynamics that place internal conversion and intersystem crossing on an equal footing,^60^ we are not aware of implementations that also account for decay by fluorescence. Since its typical time scales (from hundreds of ps to μs) are a lot longer than the ultrafast processes (typically below 1 ps) that are usually probed in nonadiabatic dynamics studies, it is safe to ignore radiative decay for most purposes. Nevertheless, as simulations advance toward longer time scales,^61^ decay by fluorescence will have to be acknowledged, and the present methodology enables exactly that. Since radiative decay is an irreversible process, the emission rate would be incorporated into the TDSE as an imaginary potential, suitable for the CS-FSSH method. The same reasoning applies to the case of phosphorescence.

Overall, our proposed methodology should largely extend the range of phenomena that can be surveyed with nonadiabatic dynamics simulations. Finally, we hope the present work is going to foment further theoretical developments on the topic.

## Conflicts of interest

There are no conflicts of interest to declare.

## Supplementary Material

SC-011-D0SC04197A-s001
