# Supplementary material for: Nonadiabatic dynamics in multidimensional complex potential energy surfaces
Source: Chem Sci. 2020 Sep 7;11(36):9827–35. doi: 10.1039/d0sc04197a (PMC8162122; doi:10.1039/d0sc04197a)
Supplement: SC-011-D0SC04197A-s001 [file SC-011-D0SC04197A-s001.pdf]

Electronic Supplementary Material (ESI) for Chemical Science.
This journal is © The Royal Society of Chemistry 2020

Electronic Supplementary Information

Nonadiabatic dynamics in multidimensional complex potential energy surfaces

Fábris Kossoski,*[a] and Mario Barbatti*[b]

[a]Aix Marseille Université, CNRS, ICR, Marseille, France. E-mail: fabris.kossoski@univ-amu.fr
[b]Aix Marseille Université, CNRS, ICR, Marseille, France. E-mail: mario.barbatti@univ-amu.fr

Contents

S1 Generalised surface hopping method 2

S2 Propagation on the model potential energy surfaces 2

S3 Computational details for the application to iodoethene 3
S3.1 Bound state calculations . . . 3
S3.2 Scattering calculations . . . 3
S3.3 Dynamics simulations . . . 3
S3.4 DEA cross section . . . 4
S3.5 Modelling the complex PES of iodoethene . . . 4

S1 Generalised surface hopping method

For an arbitrary set $\{\psi_j\}$ which is used to expand the electronic wave function (eq. 2 in the main text), the fewest switches probabilities[1] are generalised to

$$P_{j\to k} = \max\left[\frac{2\Delta t}{\rho_{jj}}\left(\frac{1}{\hbar}\mathrm{Im}(\rho_{\mathrm{kj}})\mathrm{H}^{\mathrm{R}}_{\mathrm{jk}} + \frac{1}{\hbar}\mathrm{Re}(\rho_{\mathrm{kj}})\frac{\Gamma_{\mathrm{jk}}}{2} - \mathrm{Re}(\rho_{\mathrm{kj}})\sigma^{\mathrm{NAC}}_{\mathrm{jk}}\right), 0\right]. \tag{S1}$$

When the set $\{\psi_j\}$ corresponds to the eigenstates of the real-valued electronic Hamiltonian H^{R}, then the dynamics takes place in the adiabatic representation. In this case, the adiabatic potential energy surfaces (PESs) are coupled by the nonadiabatic coupling vectors $\mathbf{F}_{jk}$. Alternatively, one could work in a basis where the nonadiabatic couplings are minimised, but then the diabatic PESs would interact via the off-diagonal diabatic couplings H_{jk}. The adiabatic representation tends to be preferred in on-the-fly trajectory methods, since the nonadiabatic couplings are more strongly peaked when compared to the smoother diabatic couplings[2]. On top of that, the adiabatic energies and nuclear gradients are directly provided when solving the electronic problem, dispensing diabatisation techniques. Therefore, in the adiabatic representation, $H^{\mathrm{R}}_{jk} = 0$ for $j \neq k$, and eq. S1 reduces to eq. 5 of the main text.

Concerning the decoherence correction, we have employed a modified version of the simplified decay of mixing[3]. At each time step, the coefficients c were updated according to

$$c'_k = c_k e^{-\Delta t/\tau_{kj}}, \forall k \neq j, \tag{S2}$$

$$c'_j = c_j\left[\frac{p - \sum_{k\neq j}|c'_k|^2}{|c_j|^2}\right]^{1/2}, \tag{S3}$$

$$\tau_{kj} = \frac{\hbar}{|E_k - E_j|}\left(1 + \frac{\alpha}{E_{\mathrm{kin}}}\right), \tag{S4}$$

where j is the current state, E_{kin} is the nuclear kinetic energy, and α is a parameter that controls the decoherence time τ_{kj}. The difference to the usual simplified decay of mixing[3] lies in eq. S3. After damping the other coefficients (eq. S2), c_j is renormalised according to the total population $p = \sum_k |c_k|^2$, which in general is different from unity.

S2 Propagation on the model potential energy surfaces

The following set of parameters was adopted for the 2REH model (defined in eqs. 6-11 of the main text): $k_0 = 3$ eV, $k_1 = 3$ eV, $R_1 = 0.25\ a_0$, $V_1(R_1) = 1$ eV, $V_2(0) = 2$ eV, $D = -1$ eV, $\alpha = 0.8\ a_0^{-1}$, $\Gamma_1(R_1) = 0.1$ eV, $\Gamma_2(0) = 0.4$ eV, $\beta = 40\ a_0^{-2}$, and a reduced mass of $\mu = 9$ amu. Weaker ($V_{12}(R_c) = 0.03$ eV) and stronger ($V_{12}(R_c) = 0.1$ eV) diabatic coupling strengths have been considered.

The adiabatic representation was employed for the propagation with the CS-FSSH method. For each initial resonant state, 1,000 trajectories were propagated, with a time step of 0.1 fs for the classical part, and of 0.005 fs for the TDSE. Initial conditions were generated by sampling from the Wigner distribution of the vibrational ground state of V_0. The fifth order Butcher's integrator[4] was employed for integrating of the TDSE, with a time step of 0.005 fs. The classical integration was performed with the velocity Verlet algorithm, with a time step of 0.1 fs. For the shorter steps, energies, nuclear velocities, nonadiabatic couplings, and resonance widths were obtained by interpolating the values computed at the longer steps of the classical propagation. After a successful hopping, the momentum was adjusted in order to conserve total energy, and for a frustrated hopping, the momentum was maintained. A decoherence correction with $\alpha = 0.1$ Hartree[5] was employed.

The quantum wavepacket was propagated on the diabatic PESs, as described elsewhere[6,7]. The propagation was performed with the split-operator technique[8], with a spacial grid of 2^{14} points, from $-2\ a_0$ to 14 a_0, and a time grid of 2^{18} points, from 0 to 500 fs.

S3 Computational details for the application to iodoethene

The description of transient anion states should be performed with either scattering methods[9–11] or adapted bound state methods[12–14]. Alternatively, usual bound state (quantum chemistry) calculations can also provide a quantitative description of such states[15–20]. The latter option must be used with caution, though, as pseudo-continuum states associated with the free electron could appear as a solution of the electronic Hamiltonian. In the present application, we have employed a mixed approach. Conventional bound state methods have been employed to compute resonance energies, albeit with a correction from scattering calculations.

S3.1 Bound state calculations

During the dynamics propagation, anion and neutral energies were computed with the multireference configuration interaction (MRCI) level of theory, with the latest implementation of the Columbus package[21]. The active space comprised 6 orbitals (the bonding σ_{CI}, two bonding π, the non-bonding n_{I}, and the antibonding π^* and σ^*_{CI} orbitals), which shared 8 electrons in the neutral and 9 in the anion. The reference wavefunction was obtained with a state-average complete active space self-consistent field calculation (SA-CASSCF) for the three lower-lying neutral singlet states in the state averaging, while considering the same (8,6) active space. We have employed the cc-pVDZ basis set[22] for carbon and hydrogen atoms, and the jorge-ADZP basis set[23] for the iodine atom, as implemented in Columbus[21,24]. The MRCI wavefunction of both anion and neutral states were obtained by accounting for single excitations from the doubly occupied orbitals (excluding the core) into the active space, and from the latter into the all virtual orbitals.

S3.2 Scattering calculations

The fixed-nuclei elastic scattering calculations were performed with the Schwinger multichannel method (SMC)[11]. The scattering wavefunction was expanded in a set of configuration state functions (CSFs), which were represented as an antisymmetrised product of a target function and a scattering orbital. Here, we have considered CSFs built from the neutral ground state (as described in the restricted Hartree-Fock approximation), and from singlet and triplet singly excited functions. Modified virtual orbitals[25] were employed to represent the unoccupied orbitals, as obtained in the field of a +8 cationic Fock operator. The CSFs were selected according to an energy criterion for the relevant orbitals[26], with a cutoff energy of 1.5 Hartree. Pseudopotentials for the iodine and carbon atoms were employed for the core electrons[27], while valence electrons were described with the same Cartesian Gaussian functions as presented elsewhere[28]. This set has been supplemented with 3s and 3p functions centred at carbon bonded to the iodine atom, with even-tempered exponents generated with a ratio of 4.

S3.3 Dynamics simulations

A development version of the Newton-X package[29,30] was employed for the dynamics simulations. In total, 1,600 trajectories were propagated, each half starting at one anion state. The initial conditions were generated by sampling from the Wigner distribution of the quantum harmonic oscillator associated with each vibrational normal mode[2,31], for a temperature of 333 K. Reasonably converged observables were attained when sampling from the Wigner distribution, differently from a previous dynamics simulation of DEA[20], where modified sampling techniques[32] were required. Newton's equations of motion were integrated with the velocity Verlet algorithm, with a time step of 0.25 fs. At each step, an additional energy calculation for the neutral ground state was performed as well, which was required to evaluate the resonance widths, as detailed in Sec. S3.5. Meanwhile, the electronic TDSE was propagated according to the fifth-order Butcher's integrator[4], with a step of 0.002 fs. For the shorter steps, energies, nuclear velocities, nonadiabatic couplings, and resonance widths were obtained by interpolating the values computed at the longer steps of the classical propagation. The time step for the TDSE is one order of magnitude shorter than what is usually employed for dynamics on real PESs, which was found to be critical for properly describing the effect of the imaginary component in the present case. When hopping between surfaces took place, nuclear mo-

menta were adjusted in the direction of the nonadiabatic coupling vector[33], and in the few trajectories that incurred into frustrated hopping, they were kept the same. The standard value of $\alpha = 0.1$ Hartree[5] was employed in the expression for the decoherence time (eq. S4). The propagation was terminated when the C−I distance reached $6a_0$, as long as at least 25 fs had passed.

S3.4 DEA cross section

In our application, we are particularly interested in computing the DEA cross section σ_k associated to each precursor anion state k, which can be computed[20,34] (in atomic units) according to

$$\sigma_k(E) = \frac{\pi}{E}\frac{1}{N_t}\sum_{i=1}^{N_t}\frac{\gamma_k(E)\Gamma^0_{k,i}}{\gamma_k(E^0_i)}g_{k,i}(E)p_{k,i}(t\to\infty). \tag{S5}$$

In the above expression, E is the electron impact energy, $p_{k,i}(t\to\infty)$ is the final population of trajectory i, and the first term in the summation denotes an energy-dependent resonance width, which is proportional to the probability of electron capture. It is comprised of the resonance width at $t = 0$, $\Gamma^0_{k,i}$, and an energy-dependent component $\gamma_k(E)$. The latter depends on the collision energy E, in the numerator, and on the resonance energy at $t = 0$, $E^0_{k,i}$, in the denominator. These functions were obtained by fitting the equilibrium-geometry eigenphase sum (obtained with the scattering calculations), according to[35]

$$\delta_k(R_0,E) = -\tan^{-1}\left(\frac{\gamma_k(E)/2}{E-\varepsilon_k-\Delta_k(E)}\right) + a_{k,0}E^{3/2} + a_{k,1}E^3, \tag{S6}$$

with

$$\gamma_k(E) = A_kE^{3/2}\exp(-b_kE), \tag{S7}$$

$$\Delta_k(E) = \frac{A_kE}{2}\left[-\frac{1}{\sqrt{\pi b_k}} + E^{1/2}e^{-b_kE}\left|\mathrm{erf}(i\sqrt{b_kE})\right|\right] - \frac{A}{4\sqrt{\pi b_k^3}}, \tag{S8}$$

where erf is the error function. The set of obtained parameters for the lower ($k = 1$) and upper ($k = 1$) anion states are shown in Tab. S1. Finally, $g_{k,i}(E)$ was represented by a normalised Gaussian function centred at the resonance energy $E^0_{k,i}$, with a line width of 0.05 eV.

k	A_k	b_k	ε_k	$a_{k,0}$	$a_{k,1}$
1	8.205435	5.408341	5.408341	1.842498	−1.990192
2	1.736950	2.017859	1.069119	−0.838433	0.397469

Table S1 Parameters of the energy-dependent functions $\gamma_1(E)$ and $\gamma_2(E)$ (for E given in eV), which appear in the expression for the DEA cross sections (eq. S5).

S3.5 Modelling the complex PES of iodoethene

Fig. S1 shows the set of results that were required in order to build the autodetachment model for the dynamics. First, it shows the elastic cross sections obtained at the neutral equilibrium geometry, as computed with the SMC scattering calculations. The peaks centred at 0.58 eV and 1.11 eV correspond to the σ^* and π^* shape resonances, respectively, which match quite well with electron transmission spectra results (0.5 eV and 1.05 eV)[36]. The scattering calculations also provide vertical resonance widths of 0.16 eV for the σ^* state and of 0.26 eV for the π^* state, corresponding to autodetachment lifetimes of 4.2 fs and 2.5 fs.

Despite the good agreement between SMC calculations and experiment, the resonance energies computed with the MRCI method are expected to be overestimated, given the well-known[10,14] difficulty in obtaining an accurate and balanced description of correlation effects for anion and neutral states. Indeed, we have obtained 2.11 eV and 3.16 eV for the σ^* and π^* states at the neutral equilibrium geometry, compared to the experimentally observed 0.5 eV and 1.05 eV[36]. Due to inaccuracies on the employed electronic structure

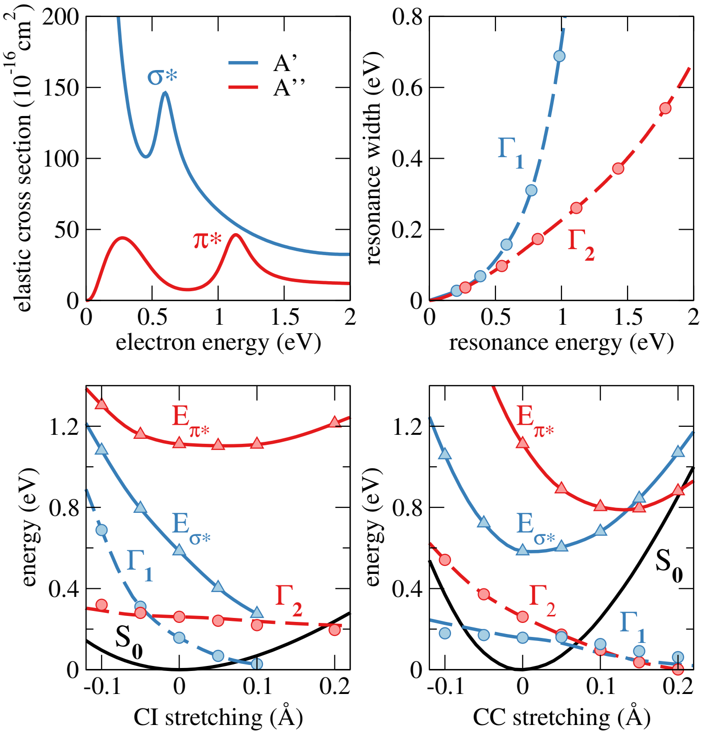

Figure S1 On the top left panel, symmetry resolved elastic cross sections, computed at the neutral equilibrium geometry. On the top right, behaviour of the model resonance widths for the lower Γ_1 and higher-lying Γ_2 anion states, as described in the text. On the bottom, PESs along the C−I and C=C stretching from the neutral equilibrium values, for the neutral, σ^* and π^* energies (triangles) and resonance widths obtained from the scattering calculations (circles) and from the model Γ_1 and Γ_2 (dashed curves).

level of theory, systematic shifts or scaling of the computed PESs are sometimes performed[20,37,38], and a similar procedure was adopted here. The resonance energy E_j associated to the anion state j was evaluated as $E_j(\mathbf{R}) = E_j^{\mathrm{MRCI}}(\mathbf{R}) + \Delta E$ where $E_j^{\mathrm{MRCI}}(\mathbf{R}) = V_j(\mathbf{R}) - V_0(\mathbf{R})$ is the MRCI resonance energy for the anion state j, given by the difference between anion V_j and neutral V_0 energies, as computed along the dynamics, and ΔE is a constant energy shift. This correction is such that the vertical ($\mathbf{R} = 0$) resonance energy E_j for the state that triggers the dynamics j matches the value obtained with the ab-initio SMC calculation $E_j^{\mathrm{SMC}}(0)$. Accordingly, when the dynamics started from the lower-lying anion state, the correction was computed as $\Delta E = E_1^{\mathrm{SMC}}(0) - E_1^{\mathrm{MRCI}}(0)$, and when the higher-lying state was initially formed, as $\Delta E = E_2^{\mathrm{SMC}}(0) - E_2^{\mathrm{MRCI}}(0)$.

We are aware that such an unbalance between neutral and anion states at the MRCI level is excessive, but as our main goal in this paper is to demonstrate the potential of the CS-FSSH methodology for dynamics simulations of real molecular systems. Moreover, we recall that this unbalance comprises a well-recognised and present challenge for electronic structure theory[10,14]. One has to move to very accurate descriptions of electronic correlation in order to achieve reasonably converged resonance energies, by accounting for triple excitations in coupled cluster methods for instance[39]. In the present case, when we include double excitations as well as the Davidson correction[40] (MRCISD+Q), the average deviation to experiment drops from 1.9 eV to 0.7 eV. Discrepancies around 0.5 eV have already been observed in calculations of resonant anions employing the MRCISD+Q[20] and the CASPT2 methodology[15–17]. Unfortunately, the MRCISD+Q level of theory does not count with analytical nuclear gradients, which is a required ingredient for the nuclei propagation. Furthermore, as our main goal in this paper is to demonstrate the use of the CS-FSSH methodology, we are satisfied that the PES shift procedure can provide a fair description of the resonances of iodoethene.

For the dynamics simulations, the imaginary PESs were modelled based on the following series of arguments. Since resonance energies and resonance widths are usually positively correlated, for both σ^*[20,41,42] and π^*[43] states, here we have assumed that the resonance widths of each state depend only on the corre-

sponding resonance energy, i.e., $\Gamma_j(\mathbf{R}) \simeq \Gamma_j[E_j(\mathbf{R})]$. Now, the resonance widths should be described more accurately in regions of the PESs where they are larger, because there the population decays more rapidly. Once the resonant anion state is formed, its energy tends to decrease, hence also the resonance energy and width. Therefore, it is more advisable to have an accurate model for the resonance widths at the beginning of the dynamics, when they are larger; than at later stages, when they would be smaller and thus of diminishing relevance to the population decay.

From a small sample of very short trajectories, the σ^* resonance was found to stabilise by C−I bond stretching, while the π^* resonance relaxed by stretching of the C=C bond. That being so, we have performed a series of scattering calculations along both stretching coordinates (the anion PESs are shown in Fig. S1), which served to build an approximated model for the widths of the lower Γ_1 and upper Γ_2 anion states, as follows. The width of the lower-lying state, Γ_1, was obtained from the behaviour of the σ^* widths along the C−I coordinate (dashed blue curve in the bottom left panel of Fig. S1). Similarly, the behaviour of the π^* width along the C=C coordinate (dashed red curve in the bottom right panel) provided the width of the higher-lying state, Γ_2. More precisely, the instantaneous resonance widths adopted for the dynamics were modelled with a third-order polynomial interpolation[44] to these pre-computed pairs of energies and widths,

$$\Gamma_j(E_j) = c_0 + c_1 E_j + c_2 E_j^2 + c_3 E_j^3. \tag{S9}$$

The set of polynomial parameter employed for each energy interval is presented in Tab. S2, and the corresponding $\Gamma_1(E_1)$ and $\Gamma_2(E_2)$ are shown in the top right panel of Fig. S1.

	Energy interval	c_0	c_1	c_2	c_3
Γ_1	[0.000000, 0.206756]	0.0000000	0.1242164	−0.0828406	0.5793206
	[0.206756, 0.385027]	0.0272616	0.1642556	0.2526995	0.6258474
	[0.385027, 0.584132]	0.0681202	0.3140227	0.5595371	0.5742861
	[0.584132, 0.771860]	0.1573580	0.6051342	0.8622322	1.3421733
	[0.771860, 0.984703]	0.3102249	1.0707663	3.2533820	0.2774268
	[0.98470, ∞)	−1.7670580	2.4933895	0.0000000	0.0000000
Γ_2	[0.000000, 0.026325]	0.0000000	0.0302233	0.3258510	5.9471457
	[0.026325, 0.272457]	0.0011299	0.0597438	0.4495795	−0.4389838
	[0.272457, 0.548139]	0.0365251	0.2012735	0.0591988	0.0223599
	[0.548139, 0.820156]	0.0969804	0.2390117	0.2513811	−0.3783485
	[0.820156, 1.112418]	0.1729807	0.2917859	0.0108890	0.0570166
	[1.112418, 1.429337]	0.2606121	0.3127613	0.1157322	0.0108206
	[1.429337, 1.786581]	0.3717004	0.3893772	0.2240029	0.0439777
	[1.786581, ∞)	−0.4702774	0.5662623	0.0000000	0.0000000

Table S2 Parameters of the polynomial interpolation (eq. S9), for energies and widths given in eV.

As additional validation of our assumptions, the profile of each Γ was checked against the coordinate that was not employed to build it in the first place. In the bottom panels of Fig. S1, dashed curves represent the model Γ_1 and Γ_2, obtained as described above, while dots are the data from the scattering calculations. We have found a good matching between Γ_1 and the σ^* widths along the C=C stretching, and also between Γ_2 and the π^* widths along the C−I stretching. This result further supports the above assumptions for the resonance widths and suggests that the conceived autodetachment model should be accurate enough for our purposes.

References

1 J. C. Tully, *J. Chem. Phys.*, 1990, **93**, 1061.

2 M. Persico and G. Granucci, *Theor. Chem. Acc.*, 2014, **133**, 1526.

3 G. Granucci and M. Persico, *J. Chem. Phys.*, 2007, **126**, 134114.

4 J. C. Butcher, *J. ACM*, 1965, **12**, 124–135.

5 C. Zhu, S. Nangia, A. W. Jasper and D. G. Truhlar, *J. Chem. Phys.*, 2004, **121**, 7658–7670.

6 H. Estrada, L. S. Cederbaum and W. Domcke, *J. Chem. Phys.*, 1986, **84**, 152.

7 C. W. McCurdy, W. A. Isaacs, H.-D. Meyer and T. N. Rescigno, *Phys. Rev. A*, 2003, **67**, 042708.

8 M. Feit, J. Fleck and A. Steiger, *J. Comput. Phys.*, 1982, **47**, 412 – 433.

9 F. A. Gianturco and A. Jain, *Phys. Rep.*, 1986, **143**, 347–425.

10 J. Tennyson, *Phys. Rep.*, 2010, **491**, 29 – 76.

11 R. F. da Costa, M. T. d. N. Varella, M. H. F. Bettega and M. A. P. Lima, *Eur. Phys. J. D*, 2015, **69**, 159.

12 G. Jolicard and E. J. Austin, *Chem. Phys. Lett.*, 1985, **121**, 106–110.

13 U. V. Riss and H. D. Meyer, *J. Phys. B: At. Mol. Opt. Phys.*, 1993, **26**, 4503–4535.

14 T.-C. Jagau, K. B. Bravaya and A. I. Krylov, *Annu. Rev. Phys. Chem.*, 2017, **68**, 525–553.

15 D. Roca-Sanjuán, M. Merchán, L. Serrano-Andrés and M. Rubio, *J. Chem. Phys.*, 2008, **129**, 095104.

16 I. González-Ramírez, J. Segarra-Martí, L. Serrano-Andrés, M. Merchán, M. Rubio and D. Roca-Sanjuán, *J. Chem. Theory Comput.*, 2012, **8**, 2769–2776.

17 A. Francés-Monerris, J. Segarra-Martí, M. Merchán and D. Roca-Sanjuán, *J. Chem. Phys.*, 2015, **143**, 0–14.

18 R. Pou-Amérigo, M. Merchán and E. Ortí, *J. Chem. Phys.*, 1999, **110**, 9536.

19 D. A. Horke, Q. Li, L. Blancafort and J. R. R. Verlet, *Nat. Chem.*, 2013, **5**, 711–7.

20 F. Kossoski, M. T. d. N. Varella and M. Barbatti, *J. Chem. Phys.*, 2019, **151**, 224104.

21 H. Lischka, R. Shepard, T. Müller, P. G. Szalay, R. M. Pitzer, A. J. A. Aquino, M. M. Araújo do Nascimento, M. Barbatti, L. T. Belcher, J.-P. Blaudeau, I. Borges, S. R. Brozell, E. A. Carter, A. Das, G. Gidofalvi, L. González, W. L. Hase, G. Kedziora, M. Kertesz, F. Kossoski, F. B. C. Machado, S. Matsika, S. A. do Monte, D. Nachtigallová, R. Nieman, M. Oppel, C. A. Parish, F. Plasser, R. F. K. Spada, E. A. Stahlberg, E. Ventura, D. R. Yarkony and Z. Zhang, *J. Chem. Phys.*, 2020, **152**, 134110.

22 T. H. Dunning, *J. Chem. Phys.*, 1989, **90**, 1007–1023.

23 P. de Oliveira, C. Barros, F. Jorge, A. Canal Neto and M. Campos, *J. Mol. Struct.*, 2010, **948**, 43 – 46.

24 H. Lischka, R. Shepard, R. M. Pitzer, I. Shavitt, M. Dallos, T. Müller, P. G. Szalay, M. Seth, G. S. Kedziora, S. Yabushita and Z. Zhang, *Phys. Chem. Chem. Phys.*, 2001, **3**, 664–673.

25 C. W. Bauschlicher, *J. Chem. Phys.*, 1980, **72**, 880.

26 F. Kossoski and M. H. F. Bettega, *J. Chem. Phys.*, 2013, **138**, 234311.

27 M. H. F. Bettega, L. G. Ferreira and M. A. P. Lima, *Phys. Rev. A*, 1993, **47**, 1111–1118.

28 F. Kossoski and M. T. D. N. Varella, *Phys. Chem. Chem. Phys.*, 2015, **17**, 17271–17278.

29 M. Barbatti, M. Ruckenbauer, F. Plasser, J. Pittner, G. Granucci, M. Persico and H. Lischka, *Wiley Interdiscip. Rev. Comput. Mol. Sci.*, 2014, **4**, 26–33.

30 Barbatti, M.; Granucci, G.; Ruckenbauer, M.; Plasser, F.; Crespo-Otero, R.; Pittner, J.; Persico, M.; Lischka, H., *NEWTON-X: A package for Newtonian dynamics close to the crossing seam. Version 2*, 2016, `http://www.newtonx.org`, accessed July 1, 2020.

31 W. B. Case, *Am. J. Phys.*, 2008, **76**, 937–946.

32 F. Kossoski and M. Barbatti, *J. Chem. Theory Comput.*, 2018, **14**, 3173–3183.

33 P. Pechukas, *Phys. Rev.*, 1969, **181**, 174–185.

34 L. Lehr and W. H. Miller, *Chem. Phys. Lett.*, 1996, **250**, 515–522.

35 P. L. Gertitschke and W. Domcke, *Phys. Rev. A*, 1993, **47**, 1031–1044.

36 J. K. Olthoff, J. A. Tossell and J. H. Moore, *J. Chem. Phys.*, 1985, **83**, 5627.

37 L. M. Frutos, T. Andruniów, F. Santoro, N. Ferré and M. Olivucci, *Proc. Natl. Acad. Sci. U. S. A.*, 2007, **104**, 7764–7769.

38 J. W. Snyder, R. M. Parrish and T. J. Martínez, *J. Phys. Chem. Lett.*, 2017, **8**, 2432–2437.

39 T.-C. Jagau, *J. Chem. Phys.*, 2018, **148**, 024104.

40 S. R. Langhoff and E. R. Davidson, *Int. J. of Quantum Chem.*, 1974, **8**, 61–72.

41 D. M. Pearl and P. D. Burrow, *J. Chem. Phys.*, 1994, **101**, 2940.

42 K. Aflatooni, G. A. Gallup and P. D. Burrow, *J. Phys. Chem. A*, 2000, **104**, 7359–7369.

43 L. M. Cornetta, K. Coutinho and M. T. d. N. Varella, *J. Chem. Phys.*, 2020, **152**, 084301.

44 H. Akima, *ACM Trans. Math. Softw.*, 1991, **17**, 341–366.
